# Down Syndrome in Brazil: Occurrence and Associated Factors

**DOI:** 10.3390/ijerph182211954

**Published:** 2021-11-14

**Authors:** Mariana Rabello Laignier, Luís Carlos Lopes-Júnior, Raquel Esperidon Santana, Franciéle Marabotti Costa Leite, Carolina Laura Brancato

**Affiliations:** 1 Nursing Department at the Health Sciences Center, Universidade Federal do Espírito Santo, Vitória 29075-910, Brazil; lopesjr.lc@gmail.com (L.C.L.-J.); francielemarabotti@gmail.com (F.M.C.L.); 2 Associação de Pais, Amigos e Pessoas com Síndrome de Down do Espírito Santo, Vitória 29075-910, Brazil; raquelesperidon@gmail.com (R.E.S.); cabrancato@hotmail.com (C.L.B.)

**Keywords:** Down syndrome, newborn, certificate of live birth, information systems, child health, epidemiology

## Abstract

Background: Down syndrome is the most frequent genetic cause of intellectual disability, with an estimated birth prevalence of 14 per 10,000 live births. In Brazil, statistical data on the occurrence of babies born with Down syndrome remain unclear. We aimed to estimate the occurrence of Down syndrome between 2012 and 2018, and to observe its association with maternal, gestational, paternal characteristics, and newborn vitality. Methods: A retrospective study was carried out using secondary data included in the Certificate of Live Birth in a state located in the southeastern region of Brazil. Data analysis was performed in the software Stata 14.1. Pearson’s chi-square test for bivariate analysis, and logistic regression for multivariate analysis were performed, with a 95% confidence interval (CI) and a significance of 5%. Results: We observed that 157 cases of Down syndrome were reported among 386,571 live births, representing an incidence of 4 in 10,000 live births. Down syndrome was associated with maternal age ≥ 35 years, paternal age ≥ 30 years, the performance of six or more prenatal consultations, prematurity, and low birth weight (*p* < 0.05). Conclusions: Women aged 35 and over were more likely to have children born with Down syndrome. In addition, there is an association of Down syndrome with premature birth, low birth weight, and the number of prenatal consultations (≥6).

## 1. Introduction

Down syndrome (DS) is a genetic disorder resulting from trisomy of chromosome 21 (whole or part), which occurs due to the failure of chromosome 21 to separate during gametogenesis, resulting in an extra chromosome in all body cells [1,2,3]. There are three main cytogenetic forms of DS: (i) free trisomy 21 consisting of a supplementary chromosome 21 in all cells [4]; (ii) mosaic trisomy 21, which has two cell lineages, one with the normal number of chromosomes and another one with an extra chromosome 21 [5], with the mechanism of occurrence consisting of an error or misdivision following fertilization during cell division; and (iii) Robertsonian translocation trisomy 21, which occurs in only 2–4% of the cases [6]. About 90% of free trisomy 21 is from a maternal meiotic error (13, 14) and only a small fraction from paternal errors [7]. Mosaic trisomy 21 occurs post-zygotically due to a malsegregation of homologs or an anaphase lag [8]. Furthermore, other forms of trisomy 21 include: (a) a terminal rearrangement of chromosome 21 around the telomeric region [9], with the final chromosome having two centromeres and satellites on both ends; and (b) a component of a double aneuploidy (for example, 48, XYY, +21 or 46, X, +21) [10,11]. Commonly, karyotype from peripheral blood is performed to confirm diagnosis for all patients suspected of DS [12,13]. The karyotype fetal DNA is one way to test for DS, through fetal cells from amniocentesis and subsequent cell culture and chromosome staining for microscope analysis [14].

DS is the most frequent genetic cause of intellectual disability, with an estimated birth prevalence of 14 per 10,000 live births [1,2,12]. Additionally, the incidence of DS increases with maternal age, and its occurrence varies in different populations (1 in 319 to 1 in 1000 live births) [6,15]. It is also known that the frequency of DS fetuses is quite high at the time of conception, but about 50% to 75% of these fetuses are lost before term [16]. Moreover, DS courses with a high biopsychosocial burden for both the individual, family members, and the health system and, consequently, represents an important public health challenge [4,5]. Children with DS have characteristic phenotypic features, delays in psychomotor development, and present an increased rate of congenital malformations [6,7]. In addition, children with DS show large variability because some have mild symptoms and complications, whereas others are more severely affected [12,16].

Patients with DS have several degrees of impairment mainly of cognitive functions, such as learning, memory, language, and executive function [1,2,3,17,18], with many experiencing difficulties in communication and understanding [19]. They may also experience emotional and behavioral challenges [20,21,22,23], which greatly impact the quality of life of these patients and of the people who care for them. As dopamine (DA) is an important neurotransmitter for cognitive function regulation, it has been pointed out that DA signaling system disturbance causes the cognitive impairments observed in DS [24,25,26,27,28]. DA systems are subject to an accelerator/brake control of their activity [29], which is pivotal for finely shaping DA responses [29,30]. Electrophysiological studies based on neuroanatomical evidence have shown that the tVTA-RMTg constitutes a major brake for DA systems: the inhibition of tVTA increases DA cell activity [31], and the stimulation of tVTA decreases it [32,33]. Additionally, DS is linked to a range of congenital anomalies, mainly cardiac, and a higher risk for respiratory, endocrine, and gastrointestinal illnesses, as well as immune-related disorders [34,35,36].

Additionally, children with DS have an increased risk of comorbidities, such as hypotonia and orofacial dysfunction, which can affect the child’s feeding ability, and hypothyroidism [37], which can potentially affect energy metabolism; autoimmunity is also increased in these patients [35]. This phenotype puts patients with DS at risk of hospitalization. This may affect their development and family relationships, and is an added disadvantage on top of their intellectual disabilities [35,36].

It has been identified that DS is the commonest known medical cause of intellectual disability [22], occurring at a rate of approximately 1 in 1000 live births worldwide [38,39,40]. Every year, about 3000 to 5000 children are born with DS [39,40]. In Brazil, statistical data on the occurrence of children born with DS remain unclear. Therefore, underreporting of statistically relevant data in Brazil is assumed. Hence, this article aimed to estimate the occurrence of DS in Brazil between 2012 and 2018, and to verify its association with the maternal, gestational, and paternal characteristics, and vitality conditions of the newborn.

## 2. Materials and Methods

An observational retrospective study was undertaken, using secondary data, generated by the Certificate of Live Birth in the Southeastern region of Brazil, between 2012 and 2018. In this study, the diagnosis of DS was described in the Certificate of Live Birth (Brazilian official document, established since June 2012, by Law No. 12,662). It is noteworthy that, through the Certificate of Live Birth, there is no possibility of knowing whether the diagnosis was made during the gestational period or after birth; thus, it may have happened by karyotype examination or phenotypic assessment. In Block VI of the Certificate of Live Birth, entitled Congenital Anomaly, field 41 indicates that all anomalies or congenital defects observed by the person responsible for the birth or by the neonatologist must be reported, without hierarchy or attempt to group them into syndromes, prioritizing the description contained in the list of codes of the International Classification of Diseases (ICD-10). Furthermore, the screening for DS made in Brazil in the first trimester is mainly the “nuchal translucency” measurement. Termination of pregnancy only due to DS diagnosis is illegal in Brazil and rates of illegal procedures are unknown. Ethical approval was obtained by the Institutional Review Board (Protocol Number 3.572.276/2019).

The primary outcome of the study was the prevalence of DS. The prevalence rate was calculated considering the number of live births with DS between 2012 and 2018, divided by the total number of live births in that same period, with the rate being expressed per 1000 births. The independent variables analyzed included: (i) “newborn”: sex (female or male), birth weight (<2500 g or ≥2500 g), and Apgar score at the first (1st) minute and fifth (5th) minute (<7 or ≥7); (ii) “mother”: maternal age (up to 34 y.o. or ≥35 y.o.), marital status (without a partner or with a partner), and education (high school, incomplete higher education, or complete higher education); and (iii) “father”: age (up to 29 y.o. or ≥30 y.o); (iv) “pregnancy and childbirth”: number of prenatal consultations (<6 or ≥6), weeks of pregnancy (<37 or ≥37 weeks), type of delivery (vaginal or cesarean). Data were analyzed using the software Statistic Data Analysis (STATA) 14.1 and presented descriptively using tables with raw frequencies (N) and relative (%) and confidence intervals (95%CI). Pearson’s chi-square test was performed for bivariate analysis. Regarding the multivariate analysis, logistic regression was performed, with entry into the model when the variables presented *p* < 0.20 and permanence with a *p*-value less than 0.05.

## 3. Results

We note that between 2012 and 2018, there were 386,571 live births, of which 157 were born with DS, representing a birth rate of 4 cases of children with DS for every 10,000 live births. Regarding the characterization of the population of cases with DS, among the 157 births, a greater proportion of newborn babies was observed, and most mothers had six or more prenatal consultations. More than half of the mothers were ≥35 years old, had incomplete higher education, and had a partner. About 84% of parents were 30 years old or older. Regarding the weeks of gestation, for 72%, they were ≥37, and in more than 90% the Apgar at the 1st minute and 5th minute was ≥7. We found that one in four births with DS weighed less than 2500 g (25.5%). Cesarean delivery was the most prevalent (68.1%) (Table 1).

Table 2 shows the prevalence of birth with DS according to the variables in our study. We verified an association between the outcome and the following variables: number of prenatal consultations, maternal age and education, paternal age, weeks of gestation, Apgar score at the 1st minute, and birth weight (*p* < 0.05).

Table 3 shows the multivariate analysis performed. We observed that after adjusting for confounding factors, DS remained associated with the number of prenatal consultations, maternal age, weeks of gestation, birth weight, and father’s age (*p* < 0.05).

We observed that pregnant women of babies with DS are 2.45 (95%CI: 1.12–5.34) more likely to have six or more prenatal consultations, when compared to the group of pregnant women with babies without DS. Likewise, women aged 35 years or older have an odds 6.0 times higher for newborns with DS. Regarding the association between DS and gestational age and birth weight, we observed that there is an approximately 2.4 times greater chance of premature births and low birth weight in the group of pregnant women whose fetus has DS, when compared to the group without this condition. In addition, older parents (30 years or older) were twice as likely to have a newborn with DS.

## 4. Discussion

We observed a prevalence of 4/10,000 cases of live births with DS through the Certificate of Live Birth, in the period between 2012 and 2018, in Brazil. A study carried out in Mexico [41] found similar results when analyzing live births in the period from 2008 to 2011 and identifying the birth rate of children with DS of 3.73 per 10,000 live births. On the contrary, a study in Argentina [42], which aimed to investigate the prevalence of DS in the period from 2009 to 2015, identified a prevalence of 17.26 per 10,000 births.

A study showed that there are significant geographic inequalities in both the total and live birth prevalence of DS [40]. For instance, in Slovenia, the prevalence of newborn infants with DS was 0.55 per 1000 births in 2012 compared to 0.51 per 1000 births in 1981, representing one of the lowest among the European countries [40]. Similar results have been reported in Hungary [18,40].

Differences in total prevalence are mainly due to the large variation in the maternal age profile of European countries an also due to the pregnancy termination rate after prenatal diagnosis of DS [40]. In addition, it was highlighted that a more than 3-fold higher live birth prevalence of DS is observed in countries with legal restrictions of termination of pregnancy after a particular gestational age [40]. In Hungary, the proportion of prenatally diagnosed cases increased from zero in 1970 to 44.3% in 1999; besides, the birth prevalence of DS decreased by 57% [43]. A study undertaken at the Paris Registry of Congenital Malformations based on 1981–2000 data showed an approximate 5% increase in the total prevalence of DS and a 3% decrease in live birth prevalence per year [40].

Research carried out in the State of Bahia, located in the Northeast region of Brazil, showed a lower percentage compared to our study, with a DS rate of 1.81 to 10,000 live births [44]. In Vale do Paraíba, in São Paulo, in 2002 and 2003, 317 live births with congenital anomalies were registered; of these, 14 cases had a diagnosis of DS [45]. However, a study carried out in the state of Mato Grosso, located in the Midwest region of Brazil, analyzed the presence of congenital anomalies among live births from 2006 to 2017 and showed a prevalence of DS of 0.25 per 1000 live births, that is, 2.5 cases per 10,000 births [46].

Two studies [47,48] have pointed out that the underreporting of congenital anomalies or genetic defects in the Certificate of Live Birth is largely due to the uncertainty of the diagnosis at the time of birth or the fear of the professional responsible for filling out the declaration, of pointing out the congenital defect in field 41—identifying DS. However, the non-notification (identification) of these cases implies the lack of planning for health actions aimed at interventions for the better quality of life of this population and their families [49,50].

In Brazil, in 2012, Law Number 12.662 [51] transformed the Certificate of Live Birth into a provisional identity document, accepted throughout the national territory. Previously used only as a form of birth registration, the Certificates of live Birth became official. The change reinforces the right of Brazilians to access public services until the Birth Certificate is registered with a notary. Additionally, according to Law 12.662, the Certificate of Live Birth shall be issued by the health professional responsible for monitoring the pregnancy, childbirth, or the newborn [51,52].

The Certificate of Live Birth is a document of great importance, which in addition to being a source of the Information System on Live Births (Sistema de Informação sobre Nascidos Vivos—SINASC), serves as basis for civil registration. The Certificate of Live Birth consists of eight blocks of information on the occurrence of births, with data related to the mother, pregnancy, delivery, and the newborn, which allows identification of the profile of live births, such as the birth weight, vitality conditions, prematurity, in addition to maternal and paternal age, among others. It should be noted that block VI, which adds only field 41, of a descriptive nature, requests that all anomalies or congenital defects observed by the person responsible for delivery or by the neonatologist who receives the child be recorded, without attempting to group them into syndromes [52]. In this respect, DS, when diagnosed during pregnancy or immediately after delivery, should be recorded in this field [52].

Regarding the association of DS with maternal characteristics, it appears that women aged ≥ 35 y.o. are 6.0 times more likely to have children born with the syndrome. This data corroborates the results from Mexico and Argentina, which show that, from the age of 35, the chance of women having a child with DS increases progressively [41,42]. Maternal age over 35 years can be considered one of the greatest risk factors for DS [53]. It is noteworthy that the process of non-disjunction can occur both in young mothers and in older-aged mothers; however, in older women, the likelihood of degradation of the meiotic process increases [53].

The average maternal age has also increased in Europe since the late 1970s. An increasing number of newborn infants with DS is expected, since the prevalence of DS is associated with maternal age. Prenatal screening and termination of affected pregnancies could counteract this effect, although this varies among countries depending on the policy, provision, and uptake of prenatal screening [18,40,54,55].

With regards to the weight, newborns with DS were 2.37 times more likely to be born with low weight than typical newborns. In line with this finding, a study published by Morris [56] presents the birth weight percentiles adjusted for sex and gestational age of typical children in relation to children with DS.

Considering 38 weeks of gestation, the average weight of newborns with DS was lower than that of normal newborns [57]. It is worth highlighting that several factors can influence intrauterine growth, such as smoking, alcohol and other drugs, arterial hypertension, chronic infectious diseases, nutritional status of the pregnant woman, short interpartum interval, high parity, maternal age (>35 years), multiple gestation, and presence of congenital anomalies. The presence of some congenital anomaly is mainly related to prematurity and delayed fetal development [58].

Regarding the gestational age, babies with DS were 2.34 times more likely to be born premature. In typical babies, the decrease in intrauterine growth is on average at 40 weeks; however, in babies with DS, the retraction of intrauterine growth can occur in the 38th week of gestation, which may be related to the higher probability of premature birth of babies with DS [56].

Prenatal diagnosis of DS can be suspected from the first trimester of pregnancy, through tests that indicate an increased risk of the fetus having congenital diseases and even accurate tests to confirm the syndrome. Nuchal translucency, an ultrasound image that assesses the accumulation of fluid in the posterior neck, performed between the 10th and 14th week of gestation, has predictive results for DS, when greater than or equal to 2.5 mm [59,60]. However, chorionic villous sampling, which analyzes the chromosomal constitution of the placenta, performed between the 10th and 12th weeks of gestation, and amniocentesis, performed after the 15th week, which examines the chromosomal constitution of the fetus, are considered accurate tests for the diagnosis of the syndrome [59,61]. Only pregnant women identified as high risk in screening tests are indicated to perform confirmatory tests [61]. The postnatal diagnosis of DS can be made through the recognition of physical characteristics (phenotype) and analysis of the newborn’s karyotype [40].

It should be noted that an invasive prenatal diagnosis, such as, CVS as well as amniocentesis, is not a feasible option for all low-risk mothers, as these procedures carry a small but finite risk and would ultimately cause more miscarriages than they would detect aneuploidy. Therefore, a number of noninvasive tests have been developed, including first-trimester risk assessment at 11 to 14 weeks, maternal serum analyte screening at 15 to 20 weeks, and sonographic fetal structural survey at 18 to 22 weeks, all of which are designed to give a woman an adjusted estimate of having an aneuploid fetus using her a priori age-related risk as a baseline. The ability to isolate fetal cells and fetal DNA from maternal blood during pregnancy has opened up exciting opportunities for improved noninvasive prenatal testing (NIPT). Direct analysis of fetal cells from maternal circulation has been challenging, given the scarcity of fetal cells in maternal blood (1:10,000–1:1,000,000). Currently, the use of cell-free DNA (cfDNA) for NIPT for chromosomal aneuploidies has been frequently reported, especially for trisomy or monosomy. Moreover, a number of commercial products are already being marketed for this indication [62].

Our study showed that mothers carrying fetuses affected with DS were more likely to have frequent prenatal consultations (OR = 2.45, CI: 1.12–5.34) secondary to the associated congenital anomalies. In contrast, a study carried out in the Northeastern region of Brazil revealed a slight difference between the group of mothers of newborns with DS and mothers of newborns without DS, in which the variable did not remain associated with the occurrence of DS [44].

The Brazilian Ministry of Health recommends that at least six prenatal consultations be carried out, preferably one in the first trimester, two in the second trimester, and three in the last trimester of pregnancy [63]. A possible justification for the high number of prenatal consultations found in this study may be related to the early identification of any congenital disorder. It is known that prenatal care has the identification of risk factors for the occurrence of congenital anomalies, which can cause maternal or fetal damage, as one of the main objectives [64].

A study held in Europe has found that the prevalence of newborn infants with DS should decrease because of prenatal screening, diagnostic tests, and pregnancy termination [3]. The experiences in Europe pointed out that the main factors influencing the prenatal detection rate of DS are the promotion of prenatal screening, keeping pregnant women informed, and the availability of DS screening to all pregnant women, with invasive diagnostic procedures offered at a younger maternal age [3,65].

Regarding the paternal age, it appears that men aged ≥ 30 y.o. are 2.0 times more likely to have children born with DS. In line with this finding is a study [66] that showed a higher frequency for the birth of children with DS in men with an average age of 31 years. Although studies of paternal meiotic non-disjunction are limited, studies have shown that paternal meiotic non-disjunction of chromosome 21 is associated with 5 to 10% of cases of trisomy 21 [7,67]. In contrast, a recent study has evidenced that advancing paternal age was not associated with an increase in risk for either DS or chromosomal disorders other than DS [68].

We recognize the limitations of our study. The main issue faced in this research was the possibility of underreporting events and the variability in the quality of data and information related to congenital anomalies in the Certificate of Live Birth, a fact that is often related to the lack of training and supervision of the professional in filling out the Certificate of Live Birth and entering this data into the system. It is also noteworthy that the scarcity of studies addressing only the profile of live births with DS makes it difficult to expand the discussion, reinforcing the present research and its findings.

## 5. Conclusions

In summary, between 2012 and 2018, there was a Down syndrome birth rate of 4 for every 10,000 live births in Brazil.

Women aged 35 and over were more likely to have children born with DS. In addition, there was an association of DS with premature birth, low birth weight, and the number of prenatal consultations (≥6). This study draws attention to the proper completion of data from the Certificate of Live Birth, attributing its real importance in the context of public health surveillance, in addition to giving visibility to the factors associated with DS. Taken together, these aspects may qualify the care delivered to the family and the newborn with DS.

## Figures and Tables

**Table 1:** Characteristics of births with Down syndrome born in Brazil for the period between 2012 and 2018 (*N* = 157).

Variables	Birth with Down Syndrome	
	*N*	%
**Sex**		
Male	75	47.8
Female	82	52.2
Number of prenatal consultations *		
<6	21	13.5
≥6	135	86.5
**Maternal age (years)**		
≤34	76	48.4
≥35	81	51.6
Level of maternal education *		
High School	25	16.0
Incomplete Higher Education	78	50.0
Complete Higher Education	53	34.0
**Marital states**		
With partner	103	65.6
Without partner	54	34.4
Father’s age (years) *		
≤29	16	15.7
≥30	86	84.3
**Weeks of gestation**		
<37	44	28.0
≥37	113	72.0
**Apgar at the 1st minute**		
<7	15	9.6
7 a 10	142	90.4
**Apgar at the 5th minute**		
<7	03	1.9
7 a 10	154	98.1
**Weight (grams)**		
<2500	40	25.5
≥2500	117	74.5
**Type of delivery**		
Vaginal	50	31.9
Cesarean	107	68.1

* Data that do not reach the total number of live births with DS (*N* = 157), as they were not filled out in the Certificate of Live Birth or were ignored.

**Table 2:** Down syndrome prevalence according to the variables of the study between 2012 and 2018 (*N* = 157).

Variables	Births with Down Syndrome		*p*-Value
	*N*	%	
**Sex**			
Male	75	0.0004	0.363
Female	82	0.0004	
Number of prenatal consultations *			
<6	21	0.0003	0.050
≥6	135	0.0004	
**Maternal age (years)**			
≤34	76	0.0002	0.000
≥35	81	0.0015	
Level of maternal education *			
High School	25	0.0003	0.000
Incomplete Higher Education	78	0.0003	
Complete Higher Education	53	0.0007	
**Marital states**			
With partner	103	0.0005	0.140
Without partner	54	0.0004	
Father’s age (years) *			
≤29	16	0.0002	0.000
≥30	86	0.0007	
**Weeks of gestation**			
<37	44	0.0012	0.000
≥37	113	0.0003	
**Apgar at the 1st minute**			
<7	15	0.0008	0.005
7 a 10	142	0.0004	
**Apgar at the 5th minute**			
<7	03	0.0007	0.343
7 a 10	154	0.0004	
**Weight (grams)**			
<2500	40	0.0013	0.000
≥2500	117	0.0003	
**Type of delivery**			
Vaginal	50	0.0004	0.296
Cesarean	107	0.0004	

* Data that do not reach the total number of live births with DS (*N* = 157), as they were not filled out in the Certificate of Live Birth or were ignored.

**Table 3:** Raw and adjusted analysis of the effects of the variables of the study on live births with Down syndrome in Brazil, between 2012 and 2018 (*N* = 157).

	Raw Analysis			Adjusted Analysis		
Variables	OR *	95%IC	*p*-Value	OR *	95%IC	*p*-Value
Number of prenatal consultations *						
<6	1.0		0.052	1.0		0.024
≥6	1.58	0.99–2.50		2.45	1.12–5.34	
**Maternal age (years)**						
≤34	1.0		0.000	1.0		0.000
≥35	6.75	4.94–9.24		6.0	4.00–9.27	
Level of maternal education *						
High School	1.0		0.000	1.0		0.196
Incomplete HE	1.18	0.75–1.85		1.48	0.74–2.96	
Complete HE	2.49	1.55–4.00		1.86	0.92–3.73	
**Marital states**						
With partner	1.28	0.92–1.78	0.141	0.76	0.48–1.20	0.239
Without partner	1.0			1.0		
**Weeks of gestation**						
<37	3.58	2.53–5.07	0.000	2.34	1.33–4.10	0.003
≥37	1.0			1.0		
**Apgar at the 1st minute**						
<7	2.12	1.25–3.62	0.006	1.57	0.77–3.20	0.212
7 a 10	1.0			1.0		
**Weight (grams)**						
<2500	3.96	2.76–5.66	0.000	2.37	1.32–4.27	0.004
≥2500	1.0			1.0		
Father’s age (years) *						
≤29	1.0		0.000	1.0		0.019
≥30	1.08	1.06–3.07		2.0	1.12–3.58	

* OR: Odds Ratio; HE: Higher Education.

## Data Availability

The data presented in this study are available on request from the corresponding author.
